# The alteration of N6-methyladenosine (m6A) modification at the transcriptome-wide level in response of heat stress in bovine mammary epithelial cells

**DOI:** 10.1186/s12864-022-09067-6

**Published:** 2022-12-14

**Authors:** Ying Qi, Yiming Zhang, Jing Zhang, Jing Wang, Qiuling Li

**Affiliations:** Hebei Key Laboratory of Animal Diversity, Langfang Key Laboratory of Cell Engineering and Application, College of Life Sciences, Langfang Normal University, Langfang, People’s Republic of China

**Keywords:** m6A, N6-methyladenosine, Heat stress, MAC-T, MeRIP-seq

## Abstract

**Background:**

Heat stress has a substantial negative economic impact on the dairy industry. N6-methyladenosine (m6A) is the most common internal RNA modification in eukaryotes and plays a key role in regulating heat stress response in animals. In dairy cows, however, this modification remains largely unexplored. Therefore, we examined the effects of heat stress on the m6A modification and gene expression in bovine mammary epithelial cells to elucidate the mechanism of heat stress response. In this study, Mammary alveolar cells-large T antigen (MAC-T) cells were incubated at 37 °C (non-heat stress group, NH) and 40 °C (heat stress group, H) for 2 hours, respectively. *HSP70*, *HSF1*, *BAX* and *CASP3* were up regulated in H group compared with those in the NH group.

**Results:**

Methylated RNA immunoprecipitation sequencing (MeRIP-seq) and RNA sequencing (RNA-seq) were conducted to identify m6A peaks and to produce gene expression data of MAC-T cells in the H and NH groups. In total, we identified 17,927 m6A peaks within 9355 genes in the H group, and 18,974 peaks within 9660 genes in the NH groups using MeRIP-seq. Compared with the NH group, 3005 significantly differentially enriched m6A peaks were identified, among which 1131 were up-regulated and 1874 were down-regulated. In addition, 1502 significantly differentially expressed genes were identified using RNA-seq, among which 796 were up-regulated and 706 were down-regulated in the H group compared to the NH group. Furthermore, 199 differentially expressed and synchronously differentially methylated genes were identified by conjoint analysis of the MeRIP-seq and RNA-seq data, which were subsequently divided into four groups: 47 hyper-up, 53 hyper-down, 59 hypo-up and 40 hypo-down genes. In addition, GO enrichment and KEGG analyses were used to analyzed the potential functions of the genes in each section.

**Conclusion:**

The comparisons of m6A modification patterns and conjoint analyses of m6A modification and gene expression profiles suggest that m6A modification plays a critical role in the heat stress response by regulating gene expression.

**Supplementary Information:**

The online version contains supplementary material available at 10.1186/s12864-022-09067-6.

## Introduction

N6-methyladenosine (m6A) is the most frequent internal RNA modification in eukaryotes, which was first discovered in the 1970s [1]. To date, over 160 types of chemical modifications have been identified in RNA molecules [2, 3], with methylation accounting for more than 60% [4]. m6A has been identified in various taxa, including yeast [5], plants [6], insects, mammals [7, 8], and some viruses [9]. m6A modification is present transcriptome-wide in more than 25% of all RNAs and preferably occurs in highly conserved regions with the consensus motif “RRACH” (R = G or A; H = A, C, or U) [10]. The methylation of “A” is highly dynamic and reversible [11]. It is catalyzed by “writers” (m6A methylases) including methyltransferase-like (METTL)3, METTL14, and Wilms’ tumor 1 associated protein (WTAP) [12, 13]. This modification can be reversed by “erasers” (m6A demethylases), including fat mass and obesity associated protein (FTO) and AlkB homolog H5 (ALKBH5) [14]. The m6A modification site is recognized by m6A “readers” (m6A binding proteins) such as YTH m6A-binding protein (YTHDF)1, YTHDF2, and YTHDF3; the heterogeneous nuclear ribonucleoprotein (HNRNP) A2/B1 and HNRNPC; and the YTH domain containing (YTHDC)1, YTHDC2, and IGF2BPs [15–17]. RNA modification through m6A may affect mRNA translation efficiency, stability, splicing, and nuclear export [4, 18–21]. Accumulating evidence indicates that m6A modification plays a vital role in cellular pathways and processes, such as cell differentiation, development, and metabolism.

Heat stress is a major limiting factor in dairy production because of its adverse effects on productivity, health, reproduction and overall welfare of dairy animals [22, 23]. The economic loss due to heat stress is estimated at over US $1.2 billion per year due to the reduction in total milk yield and the reduced quality of milk [22, 24]. The long-term increase in global temperatures aggravates the negative impacts of heat stress. And genetic antagonism between heat tolerance and milk production is an additional complicating factor [25]. Dairy cattle have higher metabolic turnover than beef cattle, and the continuous selection for higher milk production results in decreased heat tolerance [26]. Thus, there is growing interest in understanding the molecular mechanisms underlying heat stress response, and better balance of thermotolerance and high milk production traits. Milk is produced in the mammary glands, thus the effect of heat stress on gene expression is particularly important with regard to these glands. Recent large-scale transcriptome sequencing has shown that heat stress significantly changes the expression profiles of coding and noncoding RNAs in mammary glands, including mRNAs, microRNAs, long noncoding RNAs and circular RNAs [27–31]. The most extensively studied heat stress related genes are heat shock protein (HSP) genes, including *HSP110* (*HSPH*), *HSP90* (*HSPC*), *HSP70* (*HSPA*), *HSP40* (*DNAJ*), and the small *HSP* (*HSPB*) family [32]. Epigenetic changes, including DNA methylation and histone acetylation, play important roles in regulating heat stress response [33, 34]. Recently, it has been confirmed that changes in m6A modifications are closely related to heat stress response as well. In sheep, the general characteristics of m6A modification and methylation profiles altered during heat stress [35]. Further, m6A modification has been reported to be involved in the response of turbot to heat stress [36]. However, the respective mechanisms have not been investigated in dairy animals. In addition, heat stress markedly increase methylation of the 5′ untranslated region (5’UTR) of *HSP70* mRNA, which facilitates initiation of cap-independent translation of *HSP70* [18]. However, the role of m6A RNA methylation in the regulation of heat stress in dairy cattle remains unclear.

This study was conducted to investigate the effects of heat stress on m6A modification and gene expression in mammary epithelial cells to elucidate the mechanism of the heat stress response. Mammary alveolar cells-large T antigen (MAC-T) cell are an established mammary epithelial cell line that retains the phenotypic characteristics of bovine mammary epithelial cells. We incubated MAC-T cells at 37 °C (non-heat stress group, NH) or 40 °C (heat stress group, H) for 2 hours. Subsequently, we investigated the whole transcriptome m6A profile and gene expression profile in the H and NH groups using methylated RNA immunoprecipitation sequencing (MeRIP-seq) and RNA sequencing (RNA-seq), respectively. Different m6A modification and gene expression patterns were systematically analyzed to improve our understanding of the role of m6A modification in regulating the heat stress response in dairy cattle.

## Results

### Gene expression of mammary epithelial cells after heat stress

**Fig. 1:**
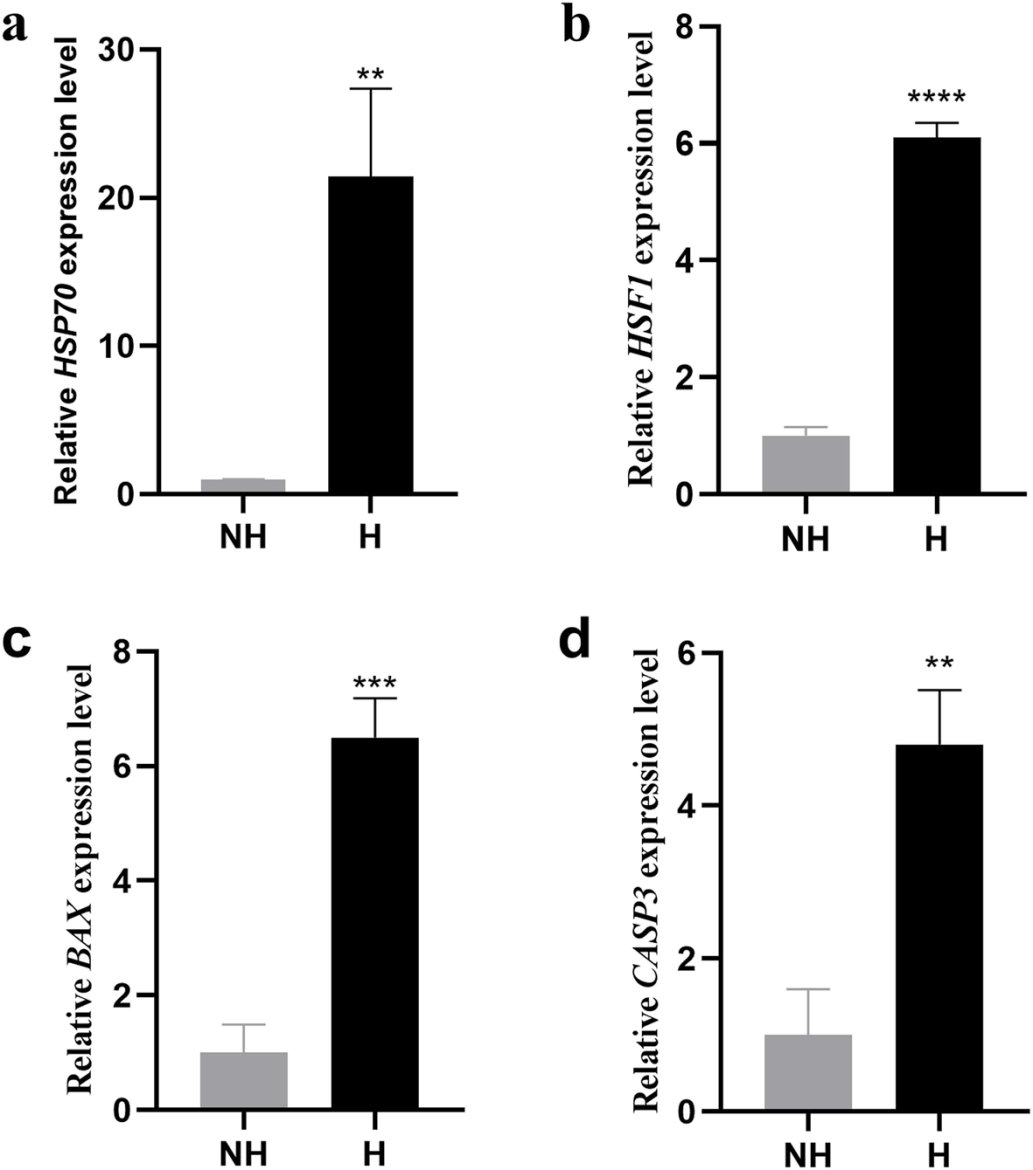
Effect of heat stress on *HSP70*, *HSF1*, *BAX*, and *CASP3* expression in MAC-T cells. Expression of *HSP70* (**a**), *HSF1* (**b**), *BAX* (**c**), and *CASP3* (**d**) was detected through qRT-PCR

MAC-T cells were cultured at 37 °C and were then assigned to two treatments, i.e., the NH treatment with, continued culturing at cultured at 37 °C, and the H treatment, with culturing at 40 °C for 2 hours. Molecular indicators of heat stress were detected by quantitative real-time PCR (qRT-PCR). The relative expression of *HSP70*, *HSF1*, *BAX* and *CASP3* was up-regulated 21.4, 6.1, 6.5, and 4.8 fold, respectively, in H group, compared to that of the NH group (Fig. 1).

### General features of m6A methylation in heat stressed MAC-T cells

To investigate the role of m6A modification due to heat stress, MeRIP-seq was performed. More than 20 M reads were generated per sample (Table 1). Over 96% of the clean reads were mapped to the reference genome, with over 94% of the clean reads being unique (Table 2). The quality control and mapping statistics of MeRIP-seq data from all samples are displayed in Tables 1 and 2, which indicated that all samples were of high quality that was sufficient for subsequent analyses.

**Table 1:** Statistics and quality control of data generated by MeRIP sequencing

Sample	Raw_reads	Clean_reads	Clean_ratio	GC_content	Q20_clean	Q30_clean
H_input	23,815,268	23,597,890	80.53%	51.92%	98.58%	95.69%
H_ip	26,437,283	26,075,056	82.47%	52.74%	98.27%	95.09%
NH_input	23,328,137	23,113,143	80.14%	52.56%	98.57%	95.75%
NH_ip	23,922,544	23,642,905	82.73%	52.35%	98.42%	95.43%

**Table 2:** Statistics of mapping data

Sample	Clean_reads	rRNA	non_rRNA	Mapped	Unique_mapped
H_Input	23,597,890	156,230(0.66%)	23,441,660	22,812,385(97.32%)	21,597,093(94.67%)
H_Ip	26,075,056	121,373(0.47%)	25,953,683	25,073,441(96.61%)	23,965,555(95.58%)
NH_Input	23,113,143	188,838(0.82%)	22,924,305	22,396,470(97.70%)	21,186,372(94.60%)
NH_Ip	23,642,905	123,226(0.52%)	23,519,679	22,818,712(97.02%)	21,736,171(95.26%)

**Fig. 2:**
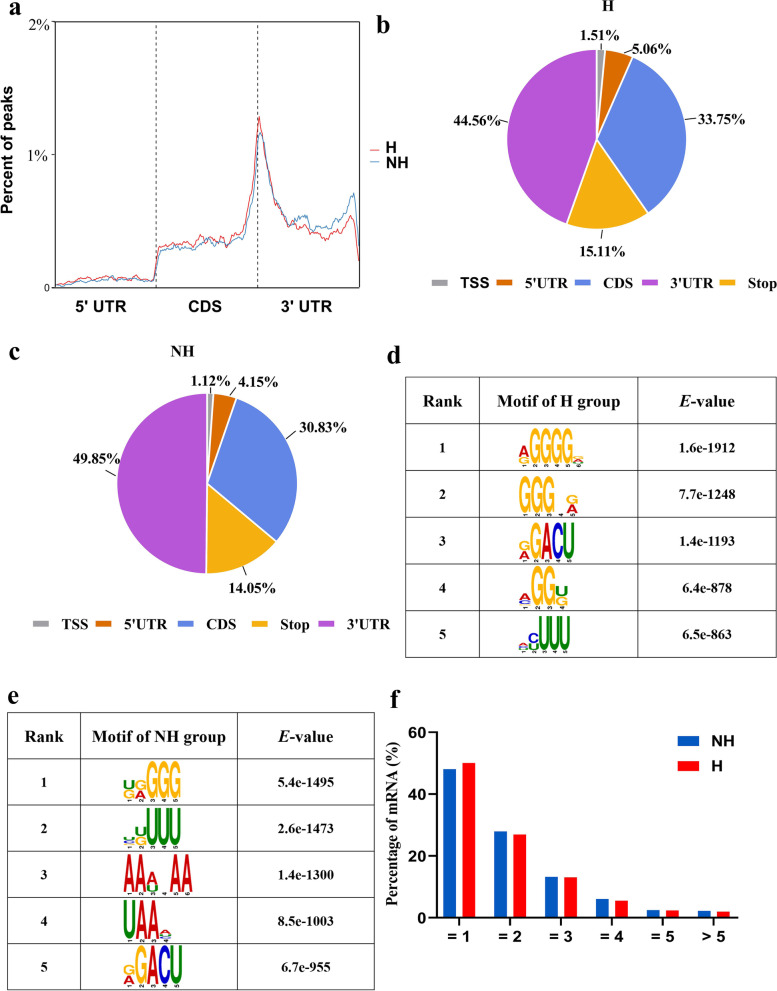
m6A topological patterns in MAC-T cells. **a** Metagene plots showing the m6A peak density distribution across the transcripts in the NH and H groups. Gene segments distribution of m6A peaks in the H (**b**) and NH (**c**) groups. The top sequence motifs enriched within m6A peaks in the H (**d**) and NH (**e**) groups. (**f**) Proportion of genes containing different numbers of m6A peaks in the NH and H groups

To investigate the distribution of m6A modifications in transcripts, we examined the metagene profiles of m6A peaks in the H and NH groups. As shown in Fig. 2a, m6A peaks were markedly enriched near the stop codon. Two further distinct peaks were observed in the start codon and 3′ UTR. The m6A density in the H group was lower than that in the NH group. To systematically assess the enrichment, the transcript was divided into five regions: transcription start site (TSS), 5′ UTR, coding sequence (CDS), 3′ UTR and the stop codon (Stop). Each m6A peak was assigned to one of the five segments, and the enrichment proportion was calculated. In both groups, more than 40% of m6A peaks were in 3′ UTR segment, more than 30% were in the CDS region, and less than 2% were at the TSS (Fig. 2b and c). The proportion of m6A peaks enriched in the TSS, 5′ UTR, CDS, and Stop segments was higher in the H group than in the NH group. However, the trend in the 3′ UTR was the opposite. The m6A consensus motif was analyzed using MEME software [37]. The top five m6A consensus motifs were in the H and NH groups are shown in Fig. 2d and e. The consensus motif “RRACH” was among the top three in the H group and among the top five in the NH group. In addition, we characterized the pattern of m6A modification of mRNA. And a unique m6A peak occurred in more than 40% of the genes. Approximately 90% of genes contained one to three m6A peaks. Only approximately 10% of the genes contained more than three peaks per gene (Fig. 2f).

### Differential m6A modification between H and NH groups

**Fig. 3:**
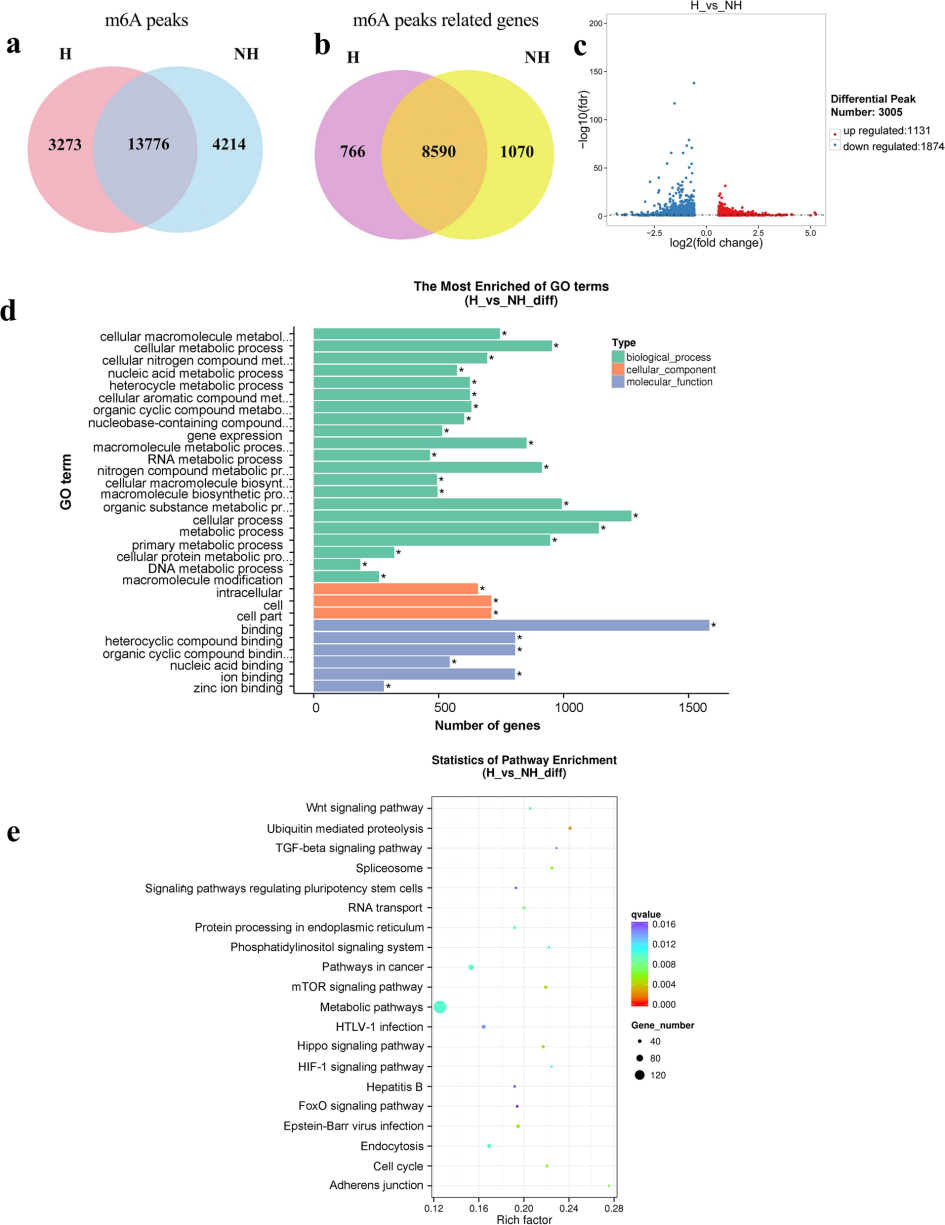
Differential m6A peaks between H and NH groups. **a** Venn diagram showing the overlap of m6A peaks between H and NH groups. **b** Venn diagram showing the overlap of m6A containing genes between H and NH groups. **c** Volcano plots of the significantly different m6A peaks. **d** GO annotation of differential m6A peak-related genes. **e** KEGG enrichment analysis of differential m6A peaks related genes

In total, we identified 17,927 m6A peaks in the H group, which were associated with 9356 genes, and 18,974 peaks in the NH group, which were associated with 9660 genes (Supplementary Tables 1 and 2). Overall, 13,776 m6A peaks overlapped between the two groups, and 3273 and 4214 peaks were unique to the H and NH group, respectively (Fig. 3a). The top 20 significantly differentially expressed m6A peaks in the H and NH groups are shown in Table 3. Moreover, 8590 m6A-containing genes overlapped between the two groups, and 766 and 1070 unique genes were unique to the in H and NH groups (Fig. 3b). As shown in Fig. 3c, we analyzed all m6A peaks and 3005 were identified as significantly differentially enriched in the H group, compared with the NH group, of which 1131 peaks were upregulated and 1874 were down regulated (*P* < 0.05, fold change > 1.5). Overall, the results of MeRIP-seq data analyses showed an altered landscape of m6A methylation under heat stressed condition.

**Table 3:** Top 20 significantly differentially expressed m6A peaks in H and NH groups

Chromosome	Gene Name	Peak Start	Peak End	*P* Value	Log2FC	Regulation
7	LOC112441507	5,739,424	5,739,544	0.011749	3.83	Up
8	KIAA2026	38,741,702	38,741,943	0.005248	3.84	Up
20	LOC104975299	71,465,294	71,465,565	0.005495	3.85	Up
11	SLC8A1	22,410,227	22,410,438	0.003548	4.08	Up
14	SMPD5	776,742	777,010	0.004786	4.08	Up
18	LOC616720	60,293,107	60,293,377	0.001698	4.1	Up
17	ZNF10	44,167,319	44,167,589	0.007079	4.11	Up
6	TBC1D1	57,214,133	57,214,284	0.045709	5	Up
16	LOC101908759	23,543,316	23,543,556	1.07E-05	5.2	Up
10	SIX4	72,876,554	72,876,734	0.001514	5.26	Up
11	DHX57	21,198,680	21,198,801	0.0002	−4.31	Down
25	LOC101902066	35,408,243	35,408,446	0.010233	−4.04	Down
18	GAN	7,992,706	7,992,827	0.002042	−3.93	Down
25	LOC107131807	2,747,385	2,747,565	0.01349	−3.91	Down
1	ZNF596	1.57E+ 08	1.57E+ 08	0.002818	−3.89	Down
X	TCEAL8	52,053,605	52,053,785	0.001622	−3.86	Down
X	PIGA	1.28E+ 08	1.28E+ 08	0.001413	−3.85	Down
1	LOC524181	45,969,243	45,969,423	0.012589	−3.85	Down
14	MYBL1	30,826,071	30,826,341	3.24E-06	−3.6	Down
4	TCAF2	1.07E+ 08	1.07E+ 08	5.5E-06	−3.57	Down

To further assess the role of m6A modification in MAC-T cells in response to heat stress, we analyzed the biological functions of differential m6A peak-related genes. All differential m6A peak-related genes were subjected to Gene Ontology (GO, http://www.geneontology.org/) and Kyoto Encyclopedia of Genes and Genomes (KEGG, http://www.kegg.jp/) pathway analyses. The top 30 significantly enriched GO items, ranked by *P*-value, are shown in Fig. 3d. In terms of biological processes, m6A-containing genes were mainly involved in metabolic processes, such as organic substance metabolic process, primary metabolic process, nitrogen compound metabolic process and cellular metabolic process. Cellular components were mainly related to intracellular part. In terms of molecular function, they were significantly correlated with heterocyclic compound binding, organic cyclic compound binding, nucleic acid binding, ion binding and zinc ion binding. The results of the KEGG analysis showed that the m6A-containing genes were significantly associated with ubiquitin mediated proteolysis, and the mammalian target of rapamycin (mTOR) signaling pathway and Hippo signaling pathway (Fig. 3e). Moreover, most genes were associated with the metabolic pathways contained.

### Conjoint analysis of MeRIP-seq and RNA-seq data

**Fig. 4:**
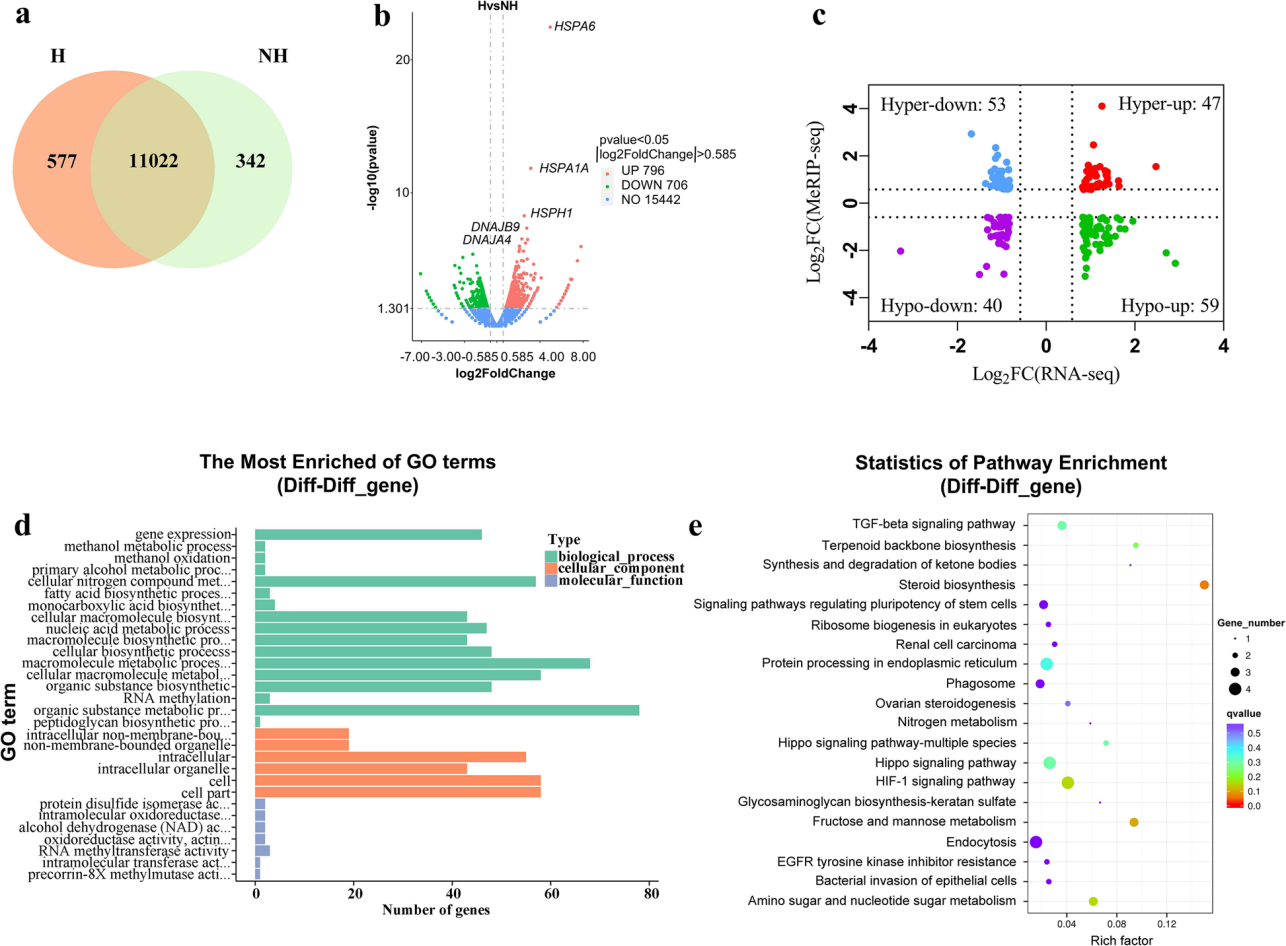
Conjoint analysis of MeRIP-seq and RNA-seq data. **a** Venn diagram showing the numbers of genes expressed in the H and NH groups. **b** Volcano plots showing differentially expressed genes. **c** Four-quadrant diagram showing the distribution of genes with significant changes in both m6A modification and mRNA expression. **d** GO annotation of genes with significant changes in m6A modification and mRNA expression. **e** KEGG enrichment analysis of genes with significant changes in m6A modification and mRNA expression

To explore the potential role of m6A modification in gene expression, we examined the transcriptome profiles of H and NH groups using RNA-seq. More genes were expressed in the H group (Fig. 4a); Compared with the NH group, 796 genes were up-regulated and 706 were down-regulated in the H group (Fig. 4b). The top 20 differentially expressed genes are listed in Table 4. Subsequently, the RNA-seq and MeRIP-seq data were combined to elucidate the relationship between m6A modification and gene expression. 199 differentially expressed genes and synchronously differentially methylated genes were observed, which are referred to here as diff-diff genes All diff-diff genes were divided into four groups, including 47 hypermethylated and up-regulated genes (hyper-up), 53 hypermethylated and down-regulated genes (hyper-down), 59 hypomethylated and up-regulated genes (hypo-up) and 40 hypomethylated and down-regulated genes (hypo-down) (Fig. 4c). All diff-diff genes were subjected to GO and KEGG analyses. The top 30 GO terms are shown in Fig. 4d. Diff-diff genes were mainly enriched with regard to biosynthetic and metabolic processes, such as organic substance biosynthetic process, cellular macromolecule biosynthetic process and cellular nitrogen compound metabolic process. The results of the KEGG analysis showed that diff-diff genes were related to steroid biosynthesis, fructose and mannose metabolism, and the HIF-1 signaling pathway (Fig. 4e).

**Table 4:** Top 20 significantly differentially expressed genes in H and NH groups

Gene Name	Regulation	Log2FC	Chromosome	Location	*P* value
HSPA6	Up	4.95	3	8,031,338...8033861	3.46E-23
HSPA1A	Up	3.16	23	27,520,317...27522790	1.42E-12
HSPH1	Up	2.54	12	29,796,074...29819631	5.24E-09
novel.144	Up	2.78	10	100,614,634...100617103	4.46E-08
DNAJB9	Up	2.33	4	59,582,765...59587977	2.88E-07
LOC104976020	Up	2.86	26	51,211,408...51219212	3.2E-07
novel.219	Up	2.66	16	45,117,300...45124664	5.95E-07
DNAJA4	Up	2.11	21	30,653,151...30669872	1.01E-06
BAG3	Up	2.09	26	39,775,458...39798895	1.04E-06
TEK	Up	7.81	8	17,050,131...17153179	1.09E-06
novel.338	Down	−2.23	29	46,702,440...46705865	4.1E-06
PLCXD1	Down	−3.15	1	88,340,913...88350182	7.2E-06
FOXS1	Down	−3.89	13	61,378,625...61379933	2.88E-05
NCBP2-AS2	Down	−1.87	1	71,410,707...71411366	3.03E-05
LOC101903988	Down	−3.48	25	3,048,814...3050919	4.3E-05
CYP2S1	Down	−1.80	18	50,379,592...50393568	0.000105
RAB6B	Down	−3.34	1	135,468,728...135526457	0.000112
LOC101904691	Down	−7.07	X	80,010,077...80024116	0.00012
FADD	Down	−1.56	29	47,373,324...47375728	0.000252
TRIP10	Down	−1.57	7	17,747,314...17757078	0.000258

### m6A modification of HSP genes

**Fig. 5:**
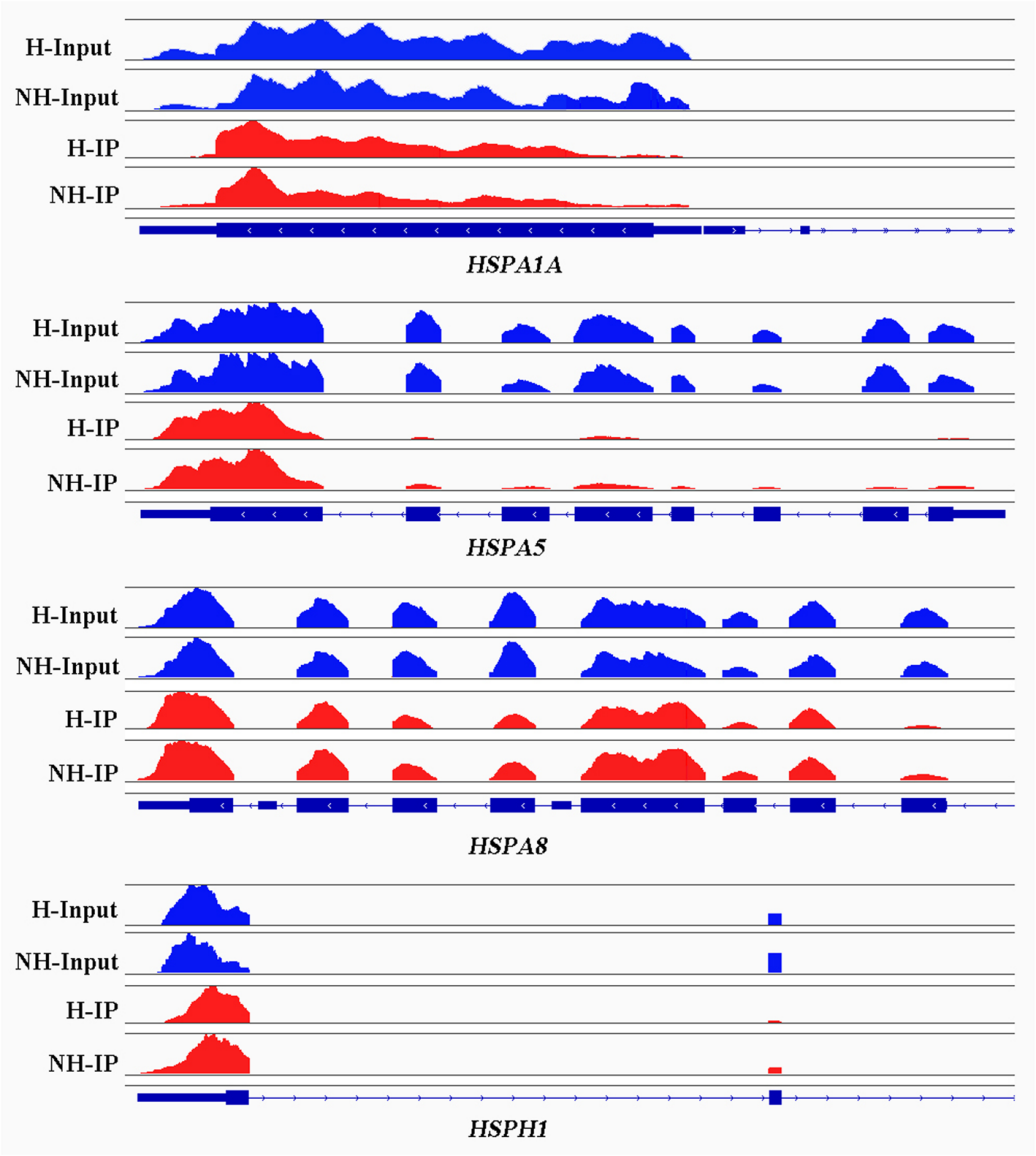
The distribution of m6A peaks in HSP mRNA. Visualization of *HSPA1A*, *HSPA5*, *HSPA8*, and *HSPH1* using Integrative Genomics Viewer (IGV) software. The blue box indicates an exon and the blue line indicates an intron

*HSP40*, *HSP70*, *HSP90*, and *HSP110* were significantly up-regulated (Table 5). The most notable m6A modification peak was observed in the vicinity of the start codon in HSP genes (Fig. 5). However, only* DNAJA1* was synchronously differentially methylated between NH and H conditions (Table 5).

**Table 5:** Expression and m6A modification of HSP genes

Gene Name	Gene expression_Log2FC	*P* value	m6A peaks_Log2FC	*P* value	HSP Family
HSPA6	4.951886251	3.46E-23	–	–	HSP70
HSPA1A	3.155571227	1.42E-12	–	–	HSP70
HSPH1	2.542206487	5.24E-09	–	–	HSP110
DNAJB9	2.325152515	2.88E-07	–	–	HSP40
DNAJA4	2.108843382	1.01E-06	–	–	HSP40
HSPA5	1.582349719	0.000166	–	–	HSP70
HSPA4L	1.672245492	0.000208	–	–	HSP70
DNAJB4	1.498594116	0.000501	–	–	HSP40
DNAJB1	1.379369553	0.001008	–	–	HSP40
DNAJA1	1.237230182	0.00306	−1.07	0.000871	HSP40
HSPA8	1.137727322	0.006158	–	–	HSP70
DNAJC12	1.219400503	0.018972	–	–	HSP40
HSP90AA1	0.94805256	0.021991	–	–	HSP90
DNAJC4	−0.925052804	0.036415	–	–	HSP40
DNAJA3	−0.872138965	0.036692	–	–	HSP40

## Discussion

Improving our understanding of the molecular mechanisms underlying heat stress response is critical for addressing the problem of heat stress in the dairy industry. m6A modification and its related enzymes have emerged as regulators of the cellular stress response [38]. Extensive research on m6A modification and cellular stress has been conducted on humans and model organisms [18, 21, 39, 40]. In *Drosophila*, m6A modification reduces the brain’s biological response to heat stress in a METTL3-dependent manner [41]. Stress granules can assemble under stress conditions to regulate mRNA translation and degradation. m6A genes are enriched in stress granules, and YTHDF proteins play essential roles in their formation in human cells [40]. However, the role of m6A modification in the heat stress response of dairy cows has not yet been elucidated. To our knowledge, this is the first transcriptome-wide study to report an m6A map of bovine mammary epithelial cells under heat stress.

We produced an m6A modification map of MAC-T cells using MeRIP-seq. According to these data, there were 1.9 peaks per gene in the H group and 2.0 peaks per gene in the NH group. The m6A modification sites were more abundant than those in sheep. As sheep genes were previously reported to contain on average 0.32 and 0.42 m6A modification sites under heat stress and control conditions, respectively [35]. Compared with the NH group, we identified 3005 significantly differentially enriched m6A peaks that were related to 2620 genes in the H group. Of these 2620 genes, 73 were zinc finger protein genes, suggesting that m6A modification may be involved in the regulation of transcription by RNA polymerase II. We found no significant difference between the H and NH groups in terms of the distribution of m6A peaks (Fig. 2a), whereas previous study found that heat shock led to a marked enrichment of m6A in the 5’UTR [21]. This discrepancy may be attributed to the use of different cell lines. In addition, we found more expressed genes but fewer m6A peaks in the H group, implying that m6A methylation may be a critical impetus for gene expression (Figs. 3 and 4a).

HSP genes are induced by cellular stress to protect cells against apoptosis and provide thermotolerance [42–44]. *HSPA1A* and *HSPA6* are the most heat-shock-inducible HSP members [45]. Consistent with previous studies, the top three up-regulated genes in the H group were *HSPA6*, *HSPA1A* and *HSPH1*. The top five included *DNAJB9*, and the top eight was *DNAJA4*, which belong to *HSP40* (*DNAJ*) family. These results indicate that the heat shock treatment of MAC-T cells was effective. Moreover, HSP genes, such as *HSPA1A*, *HSPA1B*, *HSPB1*, *HSPA9*, *HSP90AA1*, and *HSPD1*, were m6A methylated [46]. In a previous study on mouse embryonic fibroblast cells, the HSP gene *HSPA1A* showed elevated m6A modifications in the 5’UTR in response to heat stress [18]. In the present study, members of *HSP40*, *HSP70*, *HSP90* and *HSP110* were found to be m6A modified according to MeRIP-seq data. We identified significantly different m6A peaks in HSP genes including *HSPD1*, *HSPA2*, and *HSPA12A*; however, these genes were not significantly differentially expressed. Meanwhile, we identified 796 up-regulated genes by RNA-seq analysis, including HSP genes such as *HSPA6*, *HSPA1A*, and *HSPH1* (Table 5). HSP genes, especially those of the *HSP70* and *HSP40* families, were significantly up-regulated at the mRNA level, which is in accordance with previous results at the protein level [47]. Intriguingly, however, no significantly different m6A peak was found in these HSP genes except for *DNAJA1*. *DNAJA1* was hypo-up gene in our conjoint analysis of MeRIP-seq and RNA-seq data.* DNAJA1*, *HSPA1A*, *DNAJB1*, and *HSPH1* are genes encoding components of the mammalian protein disaggregation/refolding machine, which dissociates and refolds aggregated proteins [48]. All these genes were significantly up-regulated under heat shock condition. This indicates that cells enhance their capacity to combat misfolded proteins caused by high temperatures by increasing protein disaggregation/refolding.m6A modification plays a key role in regulating mRNA splicing, stability, localization and translation efficiency [18–21]. In line with previous findings, we found that the differential m6A peaks were related to biological progress items such as RNA metabolic process in GO analysis and RNA transport pathway in KEGG analysis.

Mammary gland growth, development and function are critical events that affect the lactation and reproductive performance of dairy cows. m6A genes are enriched for key signaling pathways such as Metabolic pathway, Hippo, Wnt, transforming growth factor β (TGFβ) and mTOR signaling pathways, and our KEGG analysis results were consistent with the results of previous studies [41]. Metabolic pathway was the most enriched pathway with the regard to m6A containing genes. Metabolic activity, especially carbohydrate and lipid metabolism, is decreased in mammary gland [49]. Our results suggest that m6A modification is involved in metabolic regulation under heat stress condition. The Wnt signaling pathway is associated with multiple stages of mammary development. In mammary gland, there are 10 Wnt proteins including Wnt 2,4, 5a, 5b, 6, 7b, 9a, 9b, 10b, and 16. Genes including *Wnt10b*, *Wnt5a*, *LRP5*, and *LRP6* showed different m6A peaks under H and NH conditions and were enriched in the Wnt signaling pathway, according to KEGG analysis. LRP5 and LRP6 are co-receptors for Wnt proteins. Both of which are required to respond to Wnt10b, and *Wnt10b* overexpression leads to excessive branching and precocious alveolar development [50, 51]. This implies that heat stress may affect mammary gland development through m6A regulated genes.

The mTOR signaling pathway is relevant for lactation, and it can be activated by amino acids to promote milk synthesis in mammary epithelial cells [52]. A previous study found that heat shock can alter the expression levels of genes associated with the mTOR signaling pathway, thus the amino acid metabolism and concentration in bovine mammary epithelial cells were proposed to regulate the mTOR signaling pathway to adapt to heat stress [53]. However, the specific underlying mechanisms remain to be elucidated. In the current study, the key molecules of the mTOR signaling pathway, such as *EIF4EBP1*, *EIF4E* and *TSC1*, were enriched according to the KEGG analysis of the differentially m6A peak-related genes. *EIF4EBP1* was hypo-down gene in the conjoint analysis of MeRIP-seq and RNA-seq data. This suggests that mammary epithelial cells may affect the mTOR signaling pathway through m6A modification- mediated gene regulation.

**Fig. 6:**
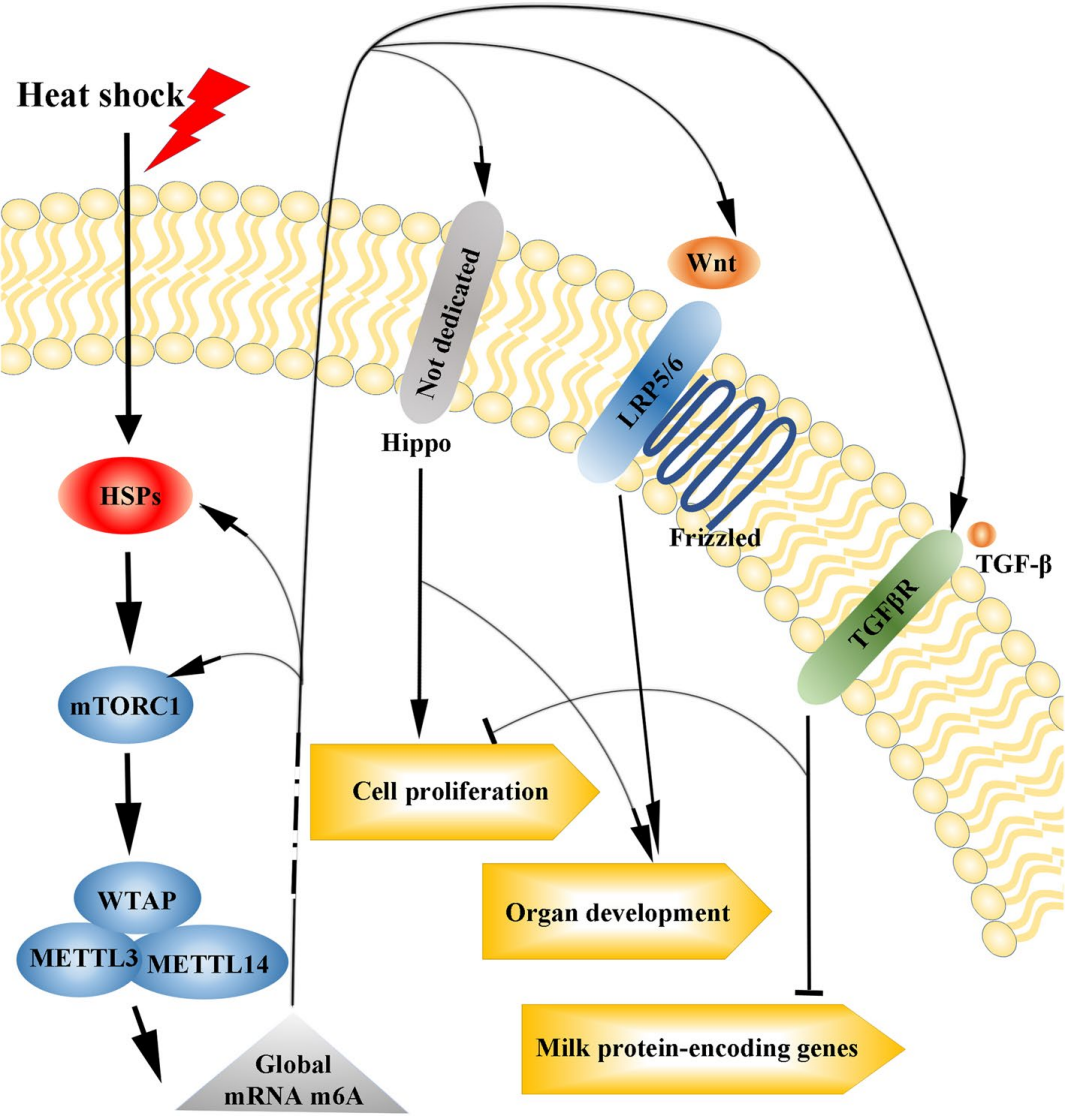
Effect of heat stress on cells through the m6A modification. The speculated molecular mechanism by which heat stress affects m6A is shown. The cell signaling pathways enriched in the KEGG analysis are indicated

Overall, we presented transcriptome-wide profiling of the m6A modification under heat stress and normal conditions and postulated a molecular mechanism that is shown in Fig. 6. HSPs, including *HSP40*, *HSP70*, *HSP90*, and *HSP110*, were up-regulated immediately after heat shock. Higher HSP expression has been associated with increased mechanistic target of rapamycin complex 1 (mTOCR1) activity [54]. mTORC1 enhances the translation of WTAP and thus activates the m6A writer complex [55]. Thereafter, the global m6A modification is increased. This can partially explain why our RNA-seq data showed that the mRNA expression levels of key m6A regulators did not change under heat stress. The m6A modification of genes associated with TGFβ, Wnt, and Hippo signaling pathways was influenced, thereby modifying cell signaling. Finally, cell proliferation was inhibited and mammary gland development was blocked during heat stress. Meanwhile, the expression of milk protein-encoding genes was reduced. These results reveal the potential regulatory mechanism of the m6A modification on milk production and milk quality under heat stress.

## Conclusion

In summary, this study provides a resource for defining the role of m6A modification in the heat stress response of dairy cattle and reveals the potential genetic regulatory mechanism of m6A modification.

## Materials and methods

### Cell culture

MAC-T cells, which is purchased from the ATCC Corporation (Beijing, China), were cultured in Dulbecco’s modified Eagle’ s medium (DMEM)/nutrient mixture F12 (Ham) medium (Gibco, Grand Island, NY, USA) supplemented with 10% fetal bovine serum (Gibco), 200 U/mL penicillin and streptomycin. Cells were cultured in T-75 cell flasks in an incubator at 37 °C and under 5% CO_2_. After 72 hours, cells were assigned to two groups, with four replicates, each. The NH group cells were continuously cultured at 37 °C and the H group at 40 °C for 2 hours.

### RNA extraction

Total RNA was isolated from MAC-T cells of the H and NH groups using TRIzol reagent (Invitrogen, Carlsbad, CA, USA), in accordance with the manufacturer’s instructions. RNA integrity and concentration were determined using an Agilent 2100 bioanalyzer (Agilent Technologies, Santa Clara, CA, USA) and a SimpliNano spectrophotometer (GE Healthcare, Freiburg, Germany), respectively.

### qRT-PCR

RevertAid First Strand cDNA Synthesis Kit (Thermo Scientific, Waltham, MA, USA) was used to reverse-transcribe the RNA samples. qRT-PCR was performed using GoTaq qPCR Master Mix (Promega, Madison, WI, USA) according to the manufacturer’s instructions. Thermocycling included 94 °C for 5 min and 35 cycles of 95 °C for 30 s, 58 °C for 15 s, and 72 °C for 30 s, and was carried out on a Bio-Rad IQ5 system (Bio-Rad, Hercules, CA, USA). β-actin was used as an internal control, and the relative gene expression level was calculated using the 2^-ΔΔCT^ method of. Primers are listed in Table 6.

**Table 6:** Primer sequences of mRNA in qRT-PCR

Gene name	Forward primer (5′-3′)	Reverse primer (5′-3′)
Bax	GCAAACTGGTGCTCAAGG	TGTGGGTGTCCCAAAGTG
CASP3	AAGATTTAGTGCCGATGC	GACCACCAAGTTCTAGGATAC
HSF1	GGCTCGCACTCCAAAGATG	GTGCTGGTGTCCACGCTG
HSPA1A	CTGAACCCGCAGAACACG	GTAGAACGCCTTGGTCTCCCCTTTG
β-actin	CATCCTGACCCTCAAGTA	CTCGTTGTAGAAGGTGTG

### MeRIP-seq and data analysis

Fragmented mRNA was incubated with anti-m6A polyclonal antibody (Synaptic Systems, Coventry, UK) in IP buffer for 2 h at 4 °C. Then, immunoprecipitated mRNA or Input were used for library construction using NEBNext Ultra II Directional RNA Library Prep Kit for Illumina (New England Biolabs, Ipswitch, MA, USA). After library quality control, sequencing was performed on an Illumina NovaSeq platform (Illumina, San Diego, CA, USA) according to standard protocols. Raw data in fastq format were processed using fastp (version 0.19.11) [56]. High-quality clean data were obtained after Q30 quality control and removal of reads containing adapter. Then, clean reads were aligned to the cattle reference genome using BWA software (v0.7.12) [57]. The R package exomePeak (v2.16.0) was used for m6A peak identification of the IP and input samples [58]. Differential peak calling was performed using R package exomePeak (v2.16.0) at *P* < 0.05 and fold change≥1.5. The m6A-enriched motifs in each group were identified by HOMER (v4.9.1). Enrichment analyses were carried out using GO and KEGG for the peak related genes [59]. GO enrichment analysis was carried out by the GOseq R package, in which gene length bias was corrected. GO terms with corrected *P* value< 0.05 were considered significantly enriched. KOBAS software (v 3.0) was used to test the statistical enrichment of genes in KEGG pathways.

### mRNA-seq

mRNA was purified from the total RNA using magnetic beads with an attached poly-T oligo. cDNA libraries were prepared using the Ultra RNA Library Prep Kit for Illumina (New England Biolabs), according to the manufacturer’s instructions. After cluster generation, the cDNA libraries were sequenced on an Illumina NovaSeq platform (Illumina), and 150 bp paired-end reads were generated. Raw data in fastq format were first processed using in-house Perl scripts, and clean data were obtained after removing reads containing adapter, poly-N, and low-quality reads. The read counts for each sample were adjusted and differential expression analysis of the H and NH groups was performed using edgeR package (v3.22.5). The *P* values were adjusted using the Benjamini and Hochberg method. Corrected *P*-value < 0.05 and fold change ≥1.5 was considered to indicate significantly differential expression.

## Supplementary Information

**Additional file 1.** m6A peaks in H and NH groups.

**Additional file 2.** m6A peaks related genes in H and NH groups.

## Data Availability

The datasets generated and analyzed during the current study are available in the NCBI Sequence Read Archive under the accession number PRJNA863056, http://www.ncbi.nlm.nih.gov/bioproject/863056.

## Abbreviations

m6A: N6-methyladenosine
NH: Non-heat stress group
H: Heat stress group
TSS: Transcription start site
5′ UTR: 5′ untranslated regions
CDS: Coding sequence
3′ UTR: 3′ untranslated regions
Stop: Stop codon
GO: Gene Ontology
KEGG: Kyoto Encyclopedia of Genes and Genomes
mTOR: Mammalian target of rapamycin
mTORC1: Mammalian target of rapamycin complex 1
TGFβ: Transforming growth factor β
